# Codetta: predicting the genetic code from nucleotide sequence

**DOI:** 10.1093/bioinformatics/btac802

**Published:** 2022-12-13

**Authors:** Yekaterina Shulgina, Sean R Eddy

**Affiliations:** Department of Molecular and Cellular Biology, Harvard University, Cambridge, MA 02138, USA; Department of Molecular and Cellular Biology, Harvard University, Cambridge, MA 02138, USA; Howard Hughes Medical Institute, Harvard University, Cambridge, MA 02138, USA

## Abstract

**Summary:**

Codetta is a Python program for predicting the genetic code table of an organism from nucleotide sequences. Codetta can analyze an arbitrary nucleotide sequence and needs no sequence annotation or taxonomic placement. The most likely amino acid decoding for each of the 64 codons is inferred from alignments of profile hidden Markov models of conserved proteins to the input sequence.

**Availability and implementation:**

Codetta 2.0 is implemented as a Python 3 program for MacOS and Linux and is available from http://eddylab.org/software/codetta/codetta2.tar.gz and at http://github.com/kshulgina/codetta.

**Supplementary information:**

Supplementary data are available at *Bioinformatics* online.

## 1 Introduction

The genetic code table defines how mRNA codons are interpreted into an amino acid sequence. Almost all of life uses the same decoding scheme; however, dozens of clades have evolved alternative genetic codes where the meaning of one or more codons is changed (Knight *et al.*, 2001). Knowing the genetic code is necessary to correctly predict protein sequences, but common practice is to assume the standard genetic code. New alternative genetic codes continue to be reported (Borges *et al.*, 2022; Shulgina and Eddy, 2021; Swart *et al.*, 2016), especially as advances in metagenomic sequencing expand known microbial diversity. Predicted proteins from newly sequenced organisms are added to protein sequence databases which primarily consist of *in silico* translations assuming a genetic code, underscoring the need for a broadly usable genetic code prediction method.

Computational methods for genetic code prediction have been developed since the early 2000s (Abascal *et al.*, 2006; Dutilh *et al.*, 2011; Mühlhausen and Kollmar, 2014; Noutahi *et al.*, 2017; Telford *et al.*, 2000). The general approach is to align the input nucleotide sequence to profiles of conserved proteins, and then, for each codon, to tally the most frequently aligned amino acid. These methods have led to the discovery of new genetic codes, but have been limited in usage due to phylogenetic constraints or error rates incompatible with large-scale analyses (Noutahi *et al.*, 2017).

Codetta is a Python program for genetic code prediction that can scale to analyze large numbers of genomes. In a recent publication, we used an early version of Codetta to screen over 250 000 bacterial and archaeal genomes and found five clades using new genetic codes (Shulgina and Eddy, 2021). Codetta improves upon previous efforts by using a probability model with a robust statistical footing to infer the amino acid for each codon and by aligning to profile hidden Markov models (HMMs) from large publicly available datasets. Here we describe Codetta 2.0, substantially revised for easier setup and usage with new options for using custom profile HMM databases and for simple parallelization on a computing cluster.

## 2 Usage

Codetta takes nucleotide sequences from a single organism as input (DNA or RNA; any level of assembly completion; no annotation needed) and predicts the genetic code from coding regions with recognizable homology. For each codon, the best amino acid meaning is selected; thus, Codetta can detect canonical stop and sense codons with new amino acid meanings (i.e. stop-to-sense and sense-to-sense reassignments). Codetta does not predict termination codons and cannot detect sense-to-stop reassignments.

Codetta performs three main steps, in order:

1. *Alignment.* A preliminary six-frame standard genetic code translation of the input sequence is aligned to profile HMMs of conserved proteins using the HMMER hmmscan program (Eddy, 2011). A typical source of profile HMMs is the Pfam database (Mistry *et al.*, 2020), but custom profile HMMs can be provided with the --profiles option. To avoid some sources of alignments that can confound genetic code inference, by default, we exclude Pfam domains that commonly pseudogenize (i.e. mobile genetic elements) or come from mitochondrial contigs; these can be optionally included.
2. *Processing.* Correspondence between codons and aligned profile HMM consensus columns is processed into a single file.
3. *Inference.* For each codon, the most likely amino acid is chosen based on a probability model of how profile HMM consensus columns match with codons in the nucleotide sequence (Shulgina and Eddy, 2021). Codetta considers 20 amino acid models and one model of non-specific translation. If an amino acid model has probability above a threshold (default 0.9999), then the amino acid meaning is selected. Otherwise, the codon is left uninferred (‘?’ output), which results from insufficient or ambiguous information. This is also the expected outcome for stop codons, which are not explicitly predicted by Codetta. Simulations show that observing a codon 10–30 times in Pfam alignments suffices to infer the correct amino acid (Shulgina and Eddy, 2021).

These three steps are bundled in the codetta.py program and are also provided as separate programs. More detail on usage options and a tutorial can be found in the associated README document.

The most computationally intensive step is the hmmscan alignment of profile HMMs. Analyzing a 4.6 Mb *Escherichia coli* genome with Pfam 35.0 as a profile HMM database takes about 30 min on a single 2.0 GHz CPU. Speed depends on the length and number of input sequences and on the size of the profile HMM database (Eddy, 2011). Codetta can parallelize the hmmscan processes across machines on a SLURM computing cluster with the --parallelize_hmmscan option.

Codetta occasionally makes incorrect predictions stemming from its underlying assumptions. Known sources of error include profile HMMs aligning to non-coding sequence, often at recent pseudogenes; extensive mRNA editing relative to the input sequence; and input sequences that differ significantly in amino acid composition from the profile HMMs (Shulgina and Eddy, 2021). Candidate new genetic codes should be validated, ideally experimentally.

## 3 Performance

Codetta was designed to predict the genetic code from nucleotide sequence for species with potentially no close taxonomic relatives. Most existing tools are clade-restricted (Abascal *et al.*, 2006; Mühlhausen and Kollmar, 2014) or require the input of a phylogenetic tree and annotated genes (Noutahi *et al.*, 2017). The only other method for arbitrary nucleotide sequences is FACIL (Dutilh *et al.*, 2011). FACIL similarly uses Pfam alignments and for each codon simply selects the most frequently aligned amino acid, filtering out low confidence predictions at a later step.

We benchmarked Codetta and FACIL on two test sets: 506 yeast nuclear genomes with three genetic codes varying at the CUG codon (13.8 Mb average genome size) and 82 mitochondrial genomes with 18 alternative genetic codes affecting 14 different codons (27 Kb average genome size). For the mitochondrial comparison, we used a custom-built profile HMM database of mitochondrial proteins to demonstrate usage of the custom profiles feature; we also repeated the Codetta analysis with Pfam. Complete results and a breakdown of per-codon prediction accuracy are provided in Supplementary Material.

**Fig. 1.**
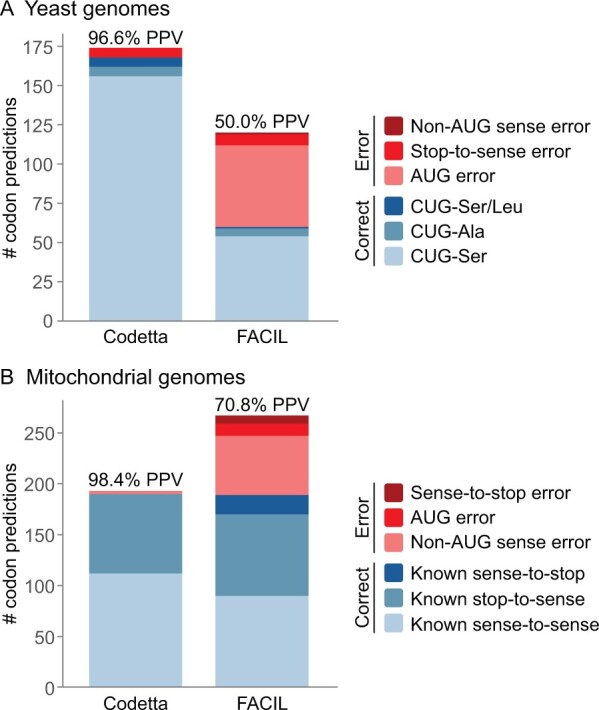
Breakdown of predicted codon reassignments for the yeast genomes test set (**A**) and mitochondrial genomes test set (**B**) with the associated PPV

Genetic code prediction requires high sensitivity to find rare codon reassignments but also a high positive predictive value (PPV) to avoid overwhelming predictions with errors. To assess how well Codetta and FACIL predict codon reassignments, we identified codon predictions that differed from the standard genetic code (predicted codon reassignments) and compared to the annotated genetic code to determine if they are correct (Fig. 1). For the yeast test set, Codetta detected almost all of the 174 reassigned CUG codons and had an overall PPV of 96.6%. The six stop-to-sense errors were likely due to the alignment of Pfam models to pseudogenes. In contrast, FACIL missed most of the true CUG reassignments and half of the predicted reassignments were errors (50.0% PPV), most commonly at AUG codons. For the mitochondrial test set, FACIL found a similar number of true reassignments but had a lower PPV (70.8%) than Codetta (98.4%). Since FACIL explicitly predicts stop codons, it was able to predict 19 true sense-to-stop reassignments missed by Codetta but also made 9 erroneous sense-to-stop predictions. In general, Codetta makes fewer sense codon errors because it leaves codons uninferred in the face of insufficient or conflicting information, which can be seen in the greater number of uninferred sense codons for mitochondria relative to FACIL (Supplementary Material). Predictions can be made less conservative by adjusting the probability threshold for predictions (-r option).

In the mitochondrial test set, Codetta predicted three sense codon reassignments that we counted as errors in Figure 1. Two green algal mitochondrial genomes had the rare isoleucine codon AUA predicted as methionine and one chytrid mitochondrial genome had the lysine codon AAG predicted as methionine. Multiple sequence alignments of mitochondrial proteins show all three of the putative reassigned codons at conserved methionine positions (Supplementary Figs S2–S4), suggesting these may be bona fide reassignments, pending additional confirmation.

## 4 Conclusion

In Shulgina and Eddy (2021), we analyzed all available bacterial and archaeal genomes, surpassing previous screens of genetic code diversity in scale. We have now extended Codetta 2.0 to be a well-documented and user-friendly tool with options to use custom profile HMM databases and to parallelize large analyses. As sequenced microbial diversity continues to grow, Codetta will allow confirming the genetic code to become a straightforward step in genome annotation, enabling discovery of new genetic codes and ensuring the accuracy of protein sequence databases.

## Supplementary Material

btac802_Supplementary_Data

## Data Availability

The data underlying this article are available in its online supplementary material.
