# Supplementary material for: Codetta: predicting the genetic code from nucleotide sequence
Source: Bioinformatics. 2022 Dec 13;39(1):btac802. doi: 10.1093/bioinformatics/btac802 (PMC9825746; doi:10.1093/bioinformatics/btac802)
Supplement: btac802_Supplementary_Data [file btac802_supplementary_data.zip › supplementary_materials.pdf]

Codetta: predicting the genetic code from nucleotide sequence–supplementary material

Yekaterina Shulgina [1] and Sean R. Eddy [1, 2]

[1]Department of Molecular and Cellular Biology, Harvard University, Cambridge, USA,
[2]Howard Hughes Medical Institute

Comparison of Codetta and FACIL codon predictions

For both the yeast genome test set and the mitochondrial genome test set, each codon in each species has a ground truth annotation of the expected translation which we compare to the predictions from Codetta and FACIL (Supplementary Files). If the translation predicted by Codetta or FACIL is consistent with the ground truth annotation, then that prediction is considered to be "correct"; if it differs from the ground truth, it is considered to be "incorrect"; and if the codon is uninferred, it is not counted as either (see Table 1). For annotated stop codons, only amino acid predictions are considered to be incorrect, since FACIL explicitly predicts stop codons while Codetta is expected to leave stop codons uninferred. Furthermore, any codon prediction that differs from the standard genetic code translation is considered to be a "predicted codon reassignment", which could be correct (a known, annotated codon reassignment) or incorrect (not consistent with the ground truth annotation) (see Table 2).

We first compared the predictions against the annotated genetic code and calculated overall rates of correct, incorrect, and uninferred codon predictions for both Codetta and FACIL (Table 1). In the yeast genome comparison (Table 1a), Codetta predicted the correct amino acid for 30,357 out of 30,366 sense codons (including reassigned CUG codons). Nine sense codons were left uninferred by Codetta, which include six cases of likely ambiguous translation of CUG and three cases of rare codon usage (*1*). No sense codons were incorrectly predicted by Codetta. In contrast, FACIL selected the incorrect amino acid for 53 sense codons (mostly AUG codons) and left 563 sense codons uninferred, including 109 reassigned CUG codons. Both methods had similar rates of incorrect stop codon predictions in both the yeast genome test set and mitochondrial genome test set.

In the mitochondrial comparison (Table 1b), FACIL had slightly more correctly predicted sense codons (4,283 (84.7%) for FACIL and 3,908 (77.2%) for Codetta); for reassigned sense codons, the number of correctly predicted codons was more even (170 for FACIL and 190 for Codetta). FACIL again had more incorrect sense codon predic-

(a) Yeast test set, predictions relative to annotated genetic code

			Codetta (Pfam)			FACIL (Pfam)		
			Correct	Incorrect	Uninferred	Correct	Incorrect	Uninferred
Sense codons	CUG	Leu (std)	332	0	0	332	0	0
		Ser	156	0	0	54	0	102
		Ala	6	0	0	5	0	1
		Ser/Leu	6 Ser	0	6	5 Leu, 1 Ser	0	6
	AUG		506	0	0	0	52	454
	All other sense codons		29,851	0	3	29,853	1	0
	Stop codons		-	6	1,512	1,510	7	1

(b) Mitochondrial test set, predictions relative to annotated genetic code

		Codetta (mito proteins)			FACIL (Pfam)		
		Correct	Incorrect	Uninferred	Correct	Incorrect	Uninferred
Sense	Sense/stop-to-sense reassign.	190	0	67	170	11	76
	AUG codons	78	0	4	29	12	41
	All other sense codons	3,640	3	1,077	4,084	55	581
Stop	Sense-to-stop reassign.	-	0	24	19	0	5
	Unchanged stops	-	0	165	119	0	46

Table 1: Per-codon comparison of Codetta and FACIL relative to the annotated genetic code on (a) 506 yeast genomes (32,384 codon predictions) and (b) 82 mitochondrial genomes (5,248 codon predictions). We show totals separately for sense codons and stop codons and further split reassigned codons and AUG (frequent FACIL error). For stop codons, only amino acid predictions are considered incorrect.

tions (78, 1.5%) compared to three for Codetta. The errors made by FACIL were distributed across reassigned sense codons, AUG codons, and other not reassigned sense codons. The three unusual sense codon predictions made by Codetta may actually be real codon reassignments, pending further support. Multiple sequence alignments of mitochondrial proteins (described in the Methods section) show that the three potentially reassigned codons occur at conserved positions for the predicted amino acid (Supplementary figures 2-4).

In the main text, we discuss the positive predictive value (PPV) for Codetta and FACIL on these two test sets. We perform these comparison by looking at predicted codon reassignments (i.e. codon predictions that differ from the *standard genetic code*) and evaluating how many of the predicted genetic codes are correct (consistent with ground truth; known codon reassignments) and how many are incorrect (inconsistent with ground truth). Table 2 shows the Codetta and FACIL predictions relative to the standard genetic code and a breakdown of the predicted codon reassignments (used to construct Figure 1).

(a) Codetta, yeast test set

**Codetta**	Prediction relative to standard code			Codon reassignments		
	Uninferred	Same	Codon reassignment	Known	Error	Codon reassignment breakdown
Standard sense	9	30,689	168	168	0	Known: 156 CUG-Ser<br>6 CUG-Ser/Leu<br>6 CUG-Ala
Standard stop	1,512	-	6	0	6	Error: 6 stop-to-sense error

(b) FACIL, yeast test set

**FACIL**	Prediction relative to standard code			Codon reassignments		
	Uninferred	Same	Codon reassignment	Known	Error	Codon reassignment breakdown
Standard sense	563	30,190	113	60	53	Known: 54 CUG-Ser<br>1 CUG-Ser/Leu<br>5 CUG-Ala<br>Error: 52 AUG error<br>1 non-AUG sense error
Standard stop	1	1,510	7	0	7	Error: 7 stop-to-sense error

(c) Codetta, mitochondrial test set

**Codetta**	Prediction relative to standard code			Codon reassignments		
	Uninferred	Same	Codon reassignment	Known	Error	Codon reassignment breakdown
Standard sense	1,169	3,718	115	112	3	Known: 112 known sense-to-sense<br>Error: 3 non-AUG sense error
Standard stop	168	-	78	78	0	Known: 78 known stop-to-sense

(d) FACIL, mitochondrial test set

**FACIL**	Prediction relative to standard code			Codon reassignments		
	Uninferred	Same	Codon reassignment	Known	Error	Codon reassignment breakdown
Standard sense	702	4,113	187	109	78	Known: 90 known sense-to-sense<br>19 known sense-to-stop<br>Error: 12 AUG error<br>58 non-AUG sense error<br>8 sense-to-stop error
Standard stop	47	119	80	80	0	Known: 80 known stop-to-sense

Table 2: Breakdown of codon inferences by Codetta and FACIL relative to the standard genetic code on the yeast genomes test set (a,b) and the mitochondrial genomes test set (c,d). Comparing relative to the standard genetic code allows us to evaluate how many of the predicted codon reassignments are known vs prediction errors, and to calculate positive predictive value (PPV). The codon reassignment breakdowns are plotted in Figure 1.

Methods

Generating test sets for performance comparison

To compare performance on the budding yeasts, we downloaded all genomes in GenBank in the class Saccharyomycetes (NCBI taxonomic identifier 4891) on December 2nd, 2021, representing 509 species (defined as unique NCBI species taxonomic IDs). Some species had multiple genome assemblies; in those cases, we sorted the assemblies by RefSeq category (reference, representative, or neither) and then by genome completeness category (complete genome, chromosome, scaffold, or contig), and randomly selected a single assembly if more than one sorted into the highest level.

We annotated the expected genetic code following the "major clade" annotation in Shen et al (2018), which is also consistent with previous studies of alternative genetic codes in yeast. Species belonging to the "major clade" CUG-Ser1 were annotated as translating CUG as serine (NCBI code 12, Alternative Yeast Nuclear Code), species in CUG-Ala were annotated as translating CUG as alanine (NCBI code 26, Pachysolen tannophilus Nuclear Code), and species in CUG-Ser2 were annotated as using both the standard genetic code and NCBI code 12 due to reported ambiguous translation of CUG (*1, 3*). All remaining species were annotated as using the standard genetic code. If a species was not included in the Shen et al (2018) analysis, then it was grouped in the same major clade as other members of its NCBI family or genus. Three species could not be placed into a major clade due to uncertain taxonomic position in NCBI annotation and were excluded from the analysis. A complete list of yeast genomes used in this analysis with the expected genetic codes can be found in Supplementary File 1.

For comparing performance on mitochondrial genomes, we collated a list of reported mitochondrial alternative genetic codes from the literature and a list of species reported to use each code. For evaluating performance, we excluded alternative genetic codes with disagreement in the literature, and those reported in only one species by a single reference. For each species, we selected a single mitochondrial genome from GenBank, picking the NCBI Reference Sequence if possible. A complete list of reported mitochondrial genetic codes and genomes used in this analysis can be found in Supplementary File 2.

Evaluating FACIL and Codetta performance

Complete results for both Codetta and FACIL can be found in Supplementary Files 3 and 4.

We compared performance against FACIL v1.0, run with default parameters (*4*). Internally, FACIL uses Pfam 22.0 as the source of profile HMMs and HMMER 3.0 for profile HMM alignment.

For the yeast analysis, we ran Codetta 2.0 with default parameters (decoding probability threshold of 0.9999) using Pfam 35.0 as the source of profile HMMs and HMMER 3.3.2 for profile HMM alignment (*5, 6*). For the mitochondrial genome analysis with Codetta, we used a lower decoding probability threshold of 0.999 to improve sensitivity and a custom profile HMM database specific to mitochondrial proteins. A Codetta analysis using Pfam 35.0 was also performed with a decoding probability threshold of 0.999 and option `-m`, which prevents exclusion of Pfam domains commonly found in mitochondrial genomes.

The custom mitochondrial protein profile HMM database was created by taking a diverse representation of mitochondrial genomes— *Homo sapiens* (NC_012920.1), *Dictyostelium discoideum* (NC_000895.1), *Tetrahymena thermophila* (NC_003029.1), *Ancoracysta twista* (NC_036491.1), *Jakoba libera* (NC_021127.1), *Andalucia godoyi* (NC_021124.1), and *Kluyveromyces lactis* (NC_006077.1)—and generating multiple sequence alignments for some of the unique annotated protein-coding genes in each genome by searching with the HMMER `jackhmmer` program against the Swiss-Prot database (UniProt release 2020_05) (*7*) with E-value inclusion thresholds tailored for each alignment. Profile HMMs were built from each alignment using the HMMER `hmmbuild` program with the `--enone` option to turn off entropy weighting. These profile HMMs were combined into a database, along with a set of Pfam domains commonly found in mitochondrial-encoded proteins. Since Codetta only considers a single aligned consensus column for each codon position in the input sequence based on alignment E-value, inclusion of redundant Pfam domains serves to ensure that the maximum amount of protein-coding sequence is aligned by one profile HMM or another. The custom mitochondrial protein profile HMM database can be obtained at `http://eddylab.org/publications/Shulgina22/mito_profiles.tar.gz`.

Generating multiple sequence alignments of unexpected mitochondrial predictions

The mitochondrial genomes of *Pseudopediastrum boryanum* (KR026342.1), *Pedinomonas minor* (NC_000892.1), and *Rhizophydium* sp. 136 (NC_003053.1) had Codetta predictions at sense codons that differed from the known genetic code. To generate multiple sequence alignments of cytochrome c oxidase subunit 1 (COX1), NADH dehydrogenase subunit 4 (ND4), and NADH dehydrogenase subunit 5 (ND5), we first found the coding regions for these genes in the genomes of interest. For NC_000892.1 and NC_003053.1, we used the coding regions provided in the genome annotation. The genome KR026342.1 had no provided annotation, so the coding regions for these genes were found by aligning the corresponding model in the custom mitochondrial profile HMM database against a 6-frame genomic translation using the HMMER `hmmsearch` program with default settings, manually examining 100 nts upstream and downstream to identify the likely start and stop codons, and checking for group I or group II

introns by aligning the region against Rfam 14.6 (*8*) using Infernal 1.1.2 program `cmscan` (*9*).

An *in silico* translation of the predicted coding sequence (using the known genetic code for each organism, codons under question translated as X) was aligned to protein sequences obtained from *Homo sapiens* (NC_012920.1), *Drosophila melanogaster* (NC_024511.2), *Arabidopsis thaliana* (NC_037304.1), *Globisporangium ultimum* (NC_014280.1), *Jakoba libera* (NC_021127.1), and *Leucocryptos marina* (NC_045933.1) using the alignment program MAFFT v7.407 with default settings (*10*). Residues implicated in human disease were annotated according to Bridges et al (2011) and Lloyd & McGeehan (2013).

These multiple sequence alignment are provided in Supplementary Files 5-7.

Supplementary figures

Supplementary figure 1. Schematic of Codetta main steps

Supplementary figure 2. Alignment of cytochrome c oxidase subunit 1 (COX1)

Supplementary figure 3. Alignment of NADH dehydrogenase subunit 4 (ND4)

Supplementary figure 4. Alignment of NADH dehydrogenase subunit 5 (ND5)

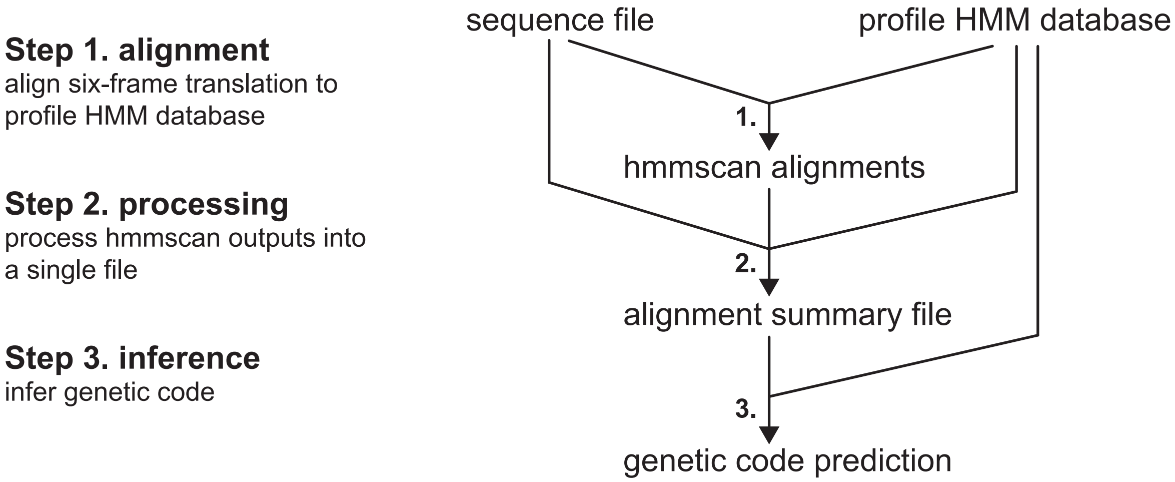

Supplementary figure 1. Schematic visualization of the main steps of Codetta, including the input and output files for each step.

```
Pboryanum_KR026342.1_COX1        MKKD--TNNLα--------------NFIYRFTCSTNAKDIGVMYLVFAAWSGVMAATMSMLIRMELSNPGPGILNGNGQL
Pminor_NC_000892.1_COX1          ------MFNL------------------KRWFFSTNHKDIGVLYLLFAGFAGVLAVTLSLLIRLQLVATGYGWLALNYQF
Rhizophydium_NC_003053.1_COX1    ------MSNLS--------------YMINRWLLSTNAKDIGVLYIIFGGFSGLVGSALSFIIRLELSGGGQIYFLGNYHD
Hsapiens_NC_012920.1_COX1        -------------------------MFADRWLFSTNHKDIGTLYLLFGAWAGVLGTALSLLIRAELGQPGN--LLGNDHI
Dmelanogaster_NC_024511.2_COX1   ---------------------------SRQWLFSTNHKDIGTLYFIFGAWAGMVGTSLSILIRAELGHPGA--LIGDDQI
Athaliana_NC_037304.1_COX1       ------MKNL------------------VRWLFSTNHKDIGTLYFIFGAIAGVMGTCFSVLIRMELARPGDQILGGNHQL
Gultimum_NC_014280.1_COX1        MNFQ-SINNWS-----------------TRWLFSTNHKDIGTLYLIFGAFSGVVGTTLSILIRMELAQPGNQIFMGNHHL
Jlibera_NC_021127.1_COX1         ------MTGL------------------TRWLYSTNHKDIGVMYIVTGAFSGILAAMISVVMRMELGLPGNQILEGNHQL
Lmarina_NC_045933.1_COX1         MAYTITYTGFSVLFLFLLFGGGGRLAFVQRWLYSTNHKDIGTLYFFFGFFSGILGTVLSILLRMELAAPGPQILPGN-QM

Pboryanum_KR026342.1_COX1        YNVLITAHGLLMLFFVVMPALMGGFGNWLVPVMIGAPDMAFPRMNNISFWVLPSSLTLLLLSSLVEQGAGVGWTAYPPLS
Pminor_NC_000892.1_COX1          YNTIVTAHGLLαVFFLLMPALαGGFGNWαVPVLIGSIDMAYPRLNNISFWLLPPSLLLLVGSMYIEαGAGTGWTVYPPLS
Rhizophydium_NC_003053.1_COX1    YNVTITGHGIVMIFFMVMPSLIGGFGNWLVPVMIGCPDMAFPRLNNVGFWLLPPSLILLI-TGLFSGGAGTGWTVYPPLS
Hsapiens_NC_012920.1_COX1        YNVIVTAHAFVMIFFMVMPIMIGGFGNWLVPLMIGAPDMAFPRMNNMSFWLLPPSLLLLLASAMVEAGAGTGWTVYPPLA
Dmelanogaster_NC_024511.2_COX1   YNVIVTAHAFIMIFFMVMPIMIGGFGNWLVPLMLGAPDMAFPRMNNMSFWLLPPALSLLLVSSMVENGAGTGWTVYPPLS
Athaliana_NC_037304.1_COX1       YNVLITAHAFLMIFFMVMPAMIGGFGNWFVPILIGAPDMAFPRLNNISFWLLPPSLLLLLSSALVEVGSGTGWTVYPPLS
Gultimum_NC_014280.1_COX1        YNVVVTAHAFIMIFFMVMPVLIGGFGNWFVPLMIGAPDMAFPRMNNISFWLLPPSLLLLVSSAIVESGAGTGWTVYPPLS
Jlibera_NC_021127.1_COX1         YNVLITAHGLLMLFWVLMPVLIGGFGNFFVPLLIGAPDMAFPRLNNISYWLLPASLLLLFFSAMVETGAGTGWTAYPPLS
Lmarina_NC_045933.1_COX1         YNAIVTAHALVMIFFFVMPTMIGGFGNWFVPIMIGAPDMAFPRMNNMSFWLLPPAIILLILSASYQGGVGTGWTVYPPLS

Pboryanum_KR026342.1_COX1        SALSHSGASVDLAIFSLHIAGAGSIMGSINFLVTVANMRAQGMTLYRLPLFVWALCFVSILLIGSLPVFAAGLTMLLTDR
Pminor_NC_000892.1_COX1          LIEFHSSASVDMAIFSLHVSGLSSLLGAINFIVTIFCMKTRGLSWRALPLFVWSVLLTAILLLLTLPVLAGSLTMLLTDR
Rhizophydium_NC_003053.1_COX1    DSAYHLGTAVDLSILSLHIAGISSLLGALNLIVTIINIRSQGLTFERLPLFVWSVKVTAWLLVLSLPVLAGAITLLLFDR
Hsapiens_NC_012920.1_COX1        GNYSHPGASVDLTIFSLHLAGVSSILGAINFITTIINMKPPAMTQYQTPLFVWSVLITAVLLLLSLPVLAAGITMLLTDR
Dmelanogaster_NC_024511.2_COX1   AGIAHGGASVDLAIFSLHLAGISSILGAVNFITTVINMRSTGISLDRMPLFVWSVVITALLLLLSLPVLAGAITMLLTDR
Athaliana_NC_037304.1_COX1       GITSHSGGAVDLAIFSLHLSGVSSILGSINFITTIFNMRGPGMTMHRLPLFVWSVLVTAFLLLLSLPVLAGAITMLLTDR
Gultimum_NC_014280.1_COX1        SVQAHSGPSVDLAIFSLHLSGISSLLGAINFLSTIYNMRAPGLSFHRLPLFVWAIFITAFLLLLTLPVLAGAITMLLTDR
Jlibera_NC_021127.1_COX1         GIQSHSGASVDLAIFSLHVSGTSSILASINFITTMLNMRAPGMNMHKMPLFCWAVFLSSFLLLLSLPVFAGGITMLLTDR
Lmarina_NC_045933.1_COX1         GLAGHPGVCVDFAIFSLHIAGASSIAGAINFIVTVYNMRCPGMFWHRVPLFVWSVFITAWLLLLSLPVLAGAITMLLTDR

Pboryanum_KR026342.1_COX1        NFNTSFFLPAGGGDVVLYQHLFWFFGHPEVYVLILPAFGVVSHVASFFA-RKPVFGYVGMVNAMAAIAVLGFLVWAHHMY
Pminor_NC_000892.1_COX1          HFNTSFYSVYGGGDPVLYQHLFWFFGHPEVYILILPAFGIISQVIαSFS-HKLMFGQLGαISAαASIGILGFLVWGHHMY
Rhizophydium_NC_003053.1_COX1    NLNTSFYDPSGGGDPILYQHLFWFFGHPEVYILILPGFGIVSHIVSRFS-QKTIFGQVGγVYAMISIGVLGFIVWSHHMY
Hsapiens_NC_012920.1_COX1        NLNTTFFDPAGGGDPILYQHLFWFFGHPEVYILILPGFGMISHIVTYYSGKKEPFGYMGMVWAMMSIGFLGFIVWAHHMF
Dmelanogaster_NC_024511.2_COX1   NLNTSFFDPAGGGDPILYQHLFWFFGHPEVYILILPGFGMISHIISQESGKKETFGSLGMIYAMLAIGLLGFIVWAHHMF
Athaliana_NC_037304.1_COX1       NFNTTFFDPAGGGDPILYQHLFWFFGHPEVYILILPGFGIISHIVSTFS-GKPVFGYLGMVYAMISIGVLGFLVWAHHMF
Gultimum_NC_014280.1_COX1        NLNTSFYDPSGGGDPVLYQHLFWFFGHPEVYVLILPAFGIVSHVTATFS-KKNVFGYLGMVYAMLSIGLLGSIVWAHHMF
Jlibera_NC_021127.1_COX1         NFNTTFFDPAGGGDPVLFQHLFWFFGHPEVYVLILPGFGIVSHIVSTFS-KKPVFGHLGMVYAMCSIGVLGSIVWAHHMY
Lmarina_NC_045933.1_COX1         NFNTCFFDYIGGGDPVTFQHLFWFFGHPEVYILILPAFGIISQVVQVFS-NKEIFGYHGMVYAMLSIGVLGFIVWAHHMY

Pboryanum_KR026342.1_COX1        TVGLDVDTRAYFTSATαIIAVPTGIKIFSWLATLYGGSLWLTTPMLFTLGFLVLFTIGGLTGIVLANAGVDIALHDTYYV
Pminor_NC_000892.1_COX1          TVGαDIDTRAYFTSATαIIAVPTSVKIFSWISTLWGGVVRLVTPLLFALGFLVLFαFGGFSGVLLANGGLDLLFHDTYYV
Rhizophydium_NC_003053.1_COX1    LVGLDIDSRAYFTAATIIIALPTGVKVFSWIATIYGGKVHYTVPMVFALGFILLFTFGGFTGVILANASIDVALHDTYYV
Hsapiens_NC_012920.1_COX1        TVGMDVDTRAYFTSATMIIAIPTGVKVFSWLATLHGSNMKWSAAVLWALGFIFLFTVGGLTGIVLANSSLDIVLHDTYYV
Dmelanogaster_NC_024511.2_COX1   TVGMDVDTRAYFTSATMIIAVPTGIKIFSWLATLHGTQLSYSPAILWALGFVFLFTVGGLTGVVLANSSVDIILHDTYYV
Athaliana_NC_037304.1_COX1       TVGLDVDTRAYFTAATMIIAVPTGIKIFSWIATMWGGSIQYKTPMLFAVGFIFLFTIGGLTGIVLANSGLDIALHDTYYV
Gultimum_NC_014280.1_COX1        TVGLDVDTRAYFSAATMIIAVPTGIKIFSWLATMWGGSLKFETPMLFTLGFLLLFVIGGVTGVIMATSGLDIAIHDTYYI
Jlibera_NC_021127.1_COX1         TVGLDVDTRAYFSAATMVIGVPTGIKVFSWLATLWGGSIELKTPMLYALGFIFLFTIGGVTGIVLSNAAIDVAMHDTYYV
Lmarina_NC_045933.1_COX1         TVGLDVDTRAYFTAATMIIAVPTGIKIFSWLATMWGGSIYLKTPMLFAVGFLFLFTFGGLTGIVLANAGIDIALHD----

Pboryanum_KR026342.1_COX1        VAHFHYVLSMGAIFGIFAAFYFWISKITGCQYPEHHGQAHFWLFFVGVNLTFFPMHFLGLAGαPRRYIDYPDAYAGWNVV
Pminor_NC_000892.1_COX1          VGHFHYVLSLGAIFALFSGFYYWTPKILGLDYDEYYAQLHFWILFVGANLTFMPQHFLGLAGαPRRIPDYPDAFSEWNVI
Rhizophydium_NC_003053.1_COX1    VGHFHYVLSLGAVLSLFAGFYYWSGKIFGYQANSKWAYVHYWVFLISINIVFFPMHFLGLQGγPRRIPDLAAGFEGWNNF
Hsapiens_NC_012920.1_COX1        VAHFHYVLSMGAVFAIMGGFIHWFPLFSGYTLDQTYAKIHFTIMFIGVNLTFFPQHFLGLSGMPRRYSDYPDAYTTWNIL
Dmelanogaster_NC_024511.2_COX1   VAHFHYVLSMGAVFAIMAGFIHWYPLFTGLTLNNKWLKSHFIIMFIGVNLTFFPQHFLGLAGMPRRYSDYPDAYTTWNIV
Athaliana_NC_037304.1_COX1       VAHFHYVLSMGAVFALFAGFYYWVGKIFGRTYPETLGQIHFWITFFGVNLTFFPMHFLGLSGMPRRIPDYPDAYAGWNAL
Gultimum_NC_014280.1_COX1        VAHFHYVLSMGAVFGIFAGFYYWIGKISGRQYPEVLGQIHFWLFFIGVNLTFFPMHFLGLAGMPRRIPDFPDAYSGWNAV
Jlibera_NC_021127.1_COX1         VGHFHYVLSLGAVFSMFAAFYYWIGKMTGYQYPETLGKLQFWLMFFGVNLTFTPMHFMGLAGMPRRIPDYPDAFAGWNLI
Lmarina_NC_045933.1_COX1         VAHFHYVLSMGAVFGLFSGFYFWIGKMTGVQYPEVLGQLHFWLTFVGVNLTFFPMHFLGLAGMPRRIPDYPDAFLGWNWV

Pboryanum_KR026342.1_COX1        ATFGSYISALSFIYFFYIVYETLANSD--RCANNPWTNEETST---------------GALEWVLPSPPAFHTYGDQLPV
Pminor_NC_000892.1_COX1          SSLGSTISVFSLIFFVLSFYSTLFKFGVYNVTSNNWLVNIFRT--------------SNSLEFLLQTPVAFHSF-RNLTV
Rhizophydium_NC_003053.1_COX1    MTLGSILTVISVILFLYLVSNALFINK---------------------RYYLM--------------PVSYSKF-INSKS
Hsapiens_NC_012920.1_COX1        SSVGSFISLTAVMLMIFMIWEAFASKR----------------------KVLMVEEPSMNLEWLYGCPPPYHTF-EE-PV
Dmelanogaster_NC_024511.2_COX1   STIGSTISLLGILFFFFIIWESLVSQR----------------------QVIYPIQLNSSIEWYQNTPPAEHSY-SELPL
Athaliana_NC_037304.1_COX1       SSFGSYISVVGICCFFVVVTITLSSGNNKRCAPSPWALELNST----------------TLEWMVQSPPAFHTF-GELPA
Gultimum_NC_014280.1_COX1        CSFGSYISFFSAILFFYIVYVTLIHGK-----------------------------------------------------
Jlibera_NC_021127.1_COX1         STYGSYLSSISVLLWLYIVYRTLTDGV--KCSNNPWAWNDTSTNQTNY----------FTLEWTLSSPPQVHTF-EEVPY
Lmarina_NC_045933.1_COX1         ASFGSYVTTIGILLFIYILYEIAFHGE--PVGKNVWENVNERTVERGFRG-------PFTLEWMIDSPPHFHTF-EELPQ

Pboryanum_KR026342.1_COX1        IRPTYQ-----LVA------------
Pminor_NC_000892.1_COX1          FNL-----------------------
Rhizophydium_NC_003053.1_COX1    FRγ-----------------------
Hsapiens_NC_012920.1_COX1        YMKS----------------------
Dmelanogaster_NC_024511.2_COX1   LTN-----------------------
Athaliana_NC_037304.1_COX1       IKETKS-----YVK------------
Gultimum_NC_014280.1_COX1        -KISK---------------------
Jlibera_NC_021127.1_COX1         VRETI-------TKH-----------
Lmarina_NC_045933.1_COX1         IRDVKNGAAFDFVRKNPGAFPLATVS
```

Supplementary figure 2. Alignment of cytochrome c oxidase subunit 1 (COX1) predicted protein sequences from the three mitochondrial genomes with an unexpected Codetta inference and six reference species. In *Pseudopediastrum boryanum* (KR026342.1) and *Pedinomonas minor* (NC_000892.1), AUA codons are represented by alphas (α). In *Rhizophydium* sp. 136 (NC_003053.1), AAG codons are represented by gammas (γ). Yellow highlighted columns contain the codons under question and are conserved for the same amino acid in all six reference species. Red triangles indicate residues located within the active site region of COX1, associated with a human disease, and reported to have a biochemical effect when mutated (from Lloyd & McGeehan, 2013).

```
Pboryanum_KR026342.1_ND4      MWFLVASL--------------------------C-------GLGAAFLANFQNαNLLAYVLSTPLVGAVILFMIPSSMT
Pminor_NC_000892.1_ND4        --MFYS----------------------------------------------------FNLFFICAFTTLLYIFYQTNKQF
Rhizophydium_NC_003053.1_ND4  -MSITSLI---------------------SFS-----------SLG------------SFLLFLFPILGIFSLWFYPQNSL
Hsapiens_NC_012920.1_ND4      ---MLKLI--------------------------------------------------VPTIMLLPLT------------
Dmelanogaster_NC_024511.2_ND4 ---MLKII--------------------------------------------------FFLLFLIPFC------------
Athaliana_NC_037304.1_ND4     --MLEHFCE-------------------------C------------YF-------NLSGLILCPVLGSIILLFIPNSRI
Gultimum_NC_014280.1_ND4      --MFNKSI--------------------------------------------------LIILLILPLTAITILSFLKNEK-
Jlibera_NC_021127.1_ND4       --MFQSNL--------------------------------------------------LPILILTPIVGIFLLFLIPSSNQ
Lmarina_NC_045933.1_ND4       -MYFNSYIMKIFYNNIFITSICLLISLFTFSSDVCLSFDSSVNLGLDYFS-----ILFLLLLFSPLISIIIISFLPEKDS

Pboryanum_KR026342.1_ND4      KAIR------------VWALTVTαITLLLCMVLWVNYTP--------CAIEPFGSVVGF-----SAGWQGLVLVRLEFGI
Pminor_NC_000892.1_ND4        TLFNWY-------NSLSIIFLLNLIYLLFST--YLNYND---------SIFαVYFFNSF---------------YYSIGα
Rhizophydium_NC_003053.1_ND4  -IIQGYEIKKIGENYIKIGLFFTLLNLLNTVILFNLYDT---------NISNLQFQLTL---FG----------KLIGGL
Hsapiens_NC_012920.1_ND4      -WLS---------KKHMIWINTTTHSLIISIIPLLFFNQINN---------------------------NLFSCSPTFSS
Dmelanogaster_NC_024511.2_ND4 -FIN-----------NMYWM-VQIMMFFISFI-FLLMNNFMN---------------------------YWSEISYFLGC
Athaliana_NC_037304.1_ND4     RLIR------------LIGLCASLITFLYSLVLWIQFDS---------STAKFQFVESL-------RWLPYENINFYLGI
Gultimum_NC_014280.1_ND4      -LIK------------NFSIFMSFFIFIISLPLWILFDK---------STSNFQYLFKI-------DWISQFNINFYLGI
Jlibera_NC_021127.1_ND4       VALK------------MTSLLFSLLTFVFSLWLWILFDE---------STSKFQFQVSY-------DWFSTSNVSATLGV
Lmarina_NC_045933.1_ND4       SLIW------------NISLTLSSFIFFLSSIIWLAFAHGNNISADFLESDLFQGVFLLIPLFGFQEWDSFLNIKPFIAI

Pboryanum_KR026342.1_ND4      DSLSLWMVLLTAALMPLAVLCSWTTαTSN----------VSAFMGLLLVLESFLFGAFTAL--DLLCFYVLYECSLLPMF
Pminor_NC_000892.1_ND4        DKFSFLFLILLSFLFSICFIYNαK------------NαIIKSKIWLFSLISαFIALLFTFISQDLLTFFIGFEFVLIPLF
Rhizophydium_NC_003053.1_ND4  DGISLWMILLVNIIIPVTILMAWKEIKLDNNPFNSASγMLKNYIFLILLVNLASIIVFFVS--DILLFYIAFEAILIPMY
Hsapiens_NC_012920.1_ND4      DPLTTPLLMLTTWLLPLTIMASQRHLSSEPLSR------KKLYLSMLISLQISLIMTFTAT--ELIMFYIFFETTLIPTL
Dmelanogaster_NC_024511.2_ND4 DMLSYGLILLSLWICSLMLLASE--MINKHNNY------KNLFLLNIIILLLLLILTFSSM--SLFMFYLFFESSLIPTL
Athaliana_NC_037304.1_ND4     DGISLFFVILTTFLIPICILVGWSGMRSY----------GKEYIIAFLICEFLMIAVFCML--DLLLFYVFFESVLIPMF
Gultimum_NC_014280.1_ND4      DGISLFFIILTTFLIPICMLISYEIINKN----------VKEYFILYFILEFCLIISFSVL--DLLIFYIFFESVLIPMF
Jlibera_NC_021127.1_ND4       DGISLFLVLLTTFLIPLCLLVSWESIKHS----------VKEYLVCFLLLESLLILIFVVL--DLVLFYVFFESVLLPMF
Lmarina_NC_045933.1_ND4       DGISILLIELTAFLIPVCFLTSISAIKTK----------IKEYIIFFFMIELLCICTFTFV--DIFLFYIFFESVLIPMF

                                                          ▼
Pboryanum_KR026342.1_ND4      LLIGLGGSRPRKVR-AAYLLVLYTLIGSLAMLPCMLLMWSQAGSTSFMLLVHES--WEPSRQL---VLWWGLFLAFAVKV
Pminor_NC_000892.1_ND4        WIVαEFGSRSNKIF-AAFFαVILTLFGSCFFLIGFFSIFLEαSSLNFNFLSFSLSYLSESRQS---FIWIFLFLGLMVKV
Rhizophydium_NC_003053.1_ND4  LLIGFYGSRNKRIENAQNTFFLYTLFGSLFMLLAIITIYIQTGSTDYQTILTLP--ISNEIQG---FLFLGFFIALAIKT
Hsapiens_NC_012920.1_ND4      AIITRWGNQPERLN-AGTYFLFYTLVGSLPLLIALIYTHNTLGSLNILLLTLTAQELSNSWANN--LMWLAYTMAFMVKM
Dmelanogaster_NC_024511.2_ND4 FLILGWGYQPERLQ-AGLYLLFYTLLVSLPMLIGIFYLMNKIGSMNFYLM--------NNFMFNYDLLYFCLLCAFLVKM
Athaliana_NC_037304.1_ND4     IIIGVWGSRQRKIK-AAYQFFLYTLLGSLFMLLAILLILFQTGTTDLQILLTTE--FSERRQI---FLWIAFFASFAVKV
Gultimum_NC_014280.1_ND4      LIIGIWGSRERKIK-ASYLFFMYTLAGSLFMFVAIIHLFLTVGTTDYQILYYSN--FDFSYEK---LYWLAFFLSFAVKV
Jlibera_NC_021127.1_ND4       IMIGVWGSRERKIR-AAYMLFLYTLLGSLFMLVAIMCIYFEAGTTDLQTLLTVD--FGERREL---LLWLAFFVSFAVKV
Lmarina_NC_045933.1_ND4       IIVGIWGSRSRKIR-AAYQFFLYTLLGSIFMFMALLYIRSVLGTTNYLILLNTMPLIDEQTQL---LLWLAFFSAFAVKV

Pboryanum_KR026342.1_ND4      PαIPVHLWLPEAHVEASTAGSVLLAGVLLKLGTYGLLRFNLSLFAYANAYYGPLIMCLSLIGLIYASLSTLRQVDLKKLV
Pminor_NC_000892.1_ND4        PLIPLHLWLPEAHGEAPTVGSVLLAGVVLKLGTYGLIRCLVFLFSNVEVFαGSILFAYGFLNMVFAALISαRQLDαKKAI
Rhizophydium_NC_003053.1_ND4  PSFPFHGWLPLAHGESSTGASVILASILLKLATYGILRFILPLFPYASNYFLPLILTLAILSIIYSCISALSLIDIKSIV
Hsapiens_NC_012920.1_ND4      PLYGLHLWLPKAHVEAPIAGSMVLAAVLLKLGGYGMMRLTLILNPLTKHMAYPFL-VLSLWGMIMTSSICLRQTDLKSLI
Dmelanogaster_NC_024511.2_ND4 PMFLVHLWLPKAHVEAPVSGSMILAGIMLKLGGYGMLRVISFLQLMNLKYSFVWI-SISLVGGVLVSLVCLRQTDLKALI
Athaliana_NC_037304.1_ND4     PMVPVHIWLPEAHVEAPTAGSVILAGILLKFGTYGFLRFSIPMFPEATLCFTPFIYTLSAIAIIYTSLTTLRQIDLKKII
Gultimum_NC_014280.1_ND4      PMLPFHVWLPEAHVEAPTAGSVILAGILLKLGSYGMVRFLIPLFPKATVYFTPYVLVLCLISVIYASLTAIRQTDLKRII
Jlibera_NC_021127.1_ND4       PMLPFHIWLPEAHVEAPTAGSVLLAGVLLKLGGYGLIRFSLGMFPDASSFFSPLVFTMSILAIIYTSLTTLRQIDLKRII
Lmarina_NC_045933.1_ND4       PMIPFHLWLPEAHVEAPTAGSVILAGILLKLGTYGFLRFSVPLFPVSSFFFMPFIFLLCSLGSIYASLTTLRQVDLKKII

                                                                                   ▼
Pboryanum_KR026342.1_ND4      AYSSIAHMSMVTLALFTMSDLGLIAASFLMLAHGIASPALFLCVGALYDRTHTKALKYLGGAATAαPLFSIMLFIFSLCN
Pminor_NC_000892.1_ND4        AYGSIAHαGTVVIGLSEVFNFSEFGSVαIαIFHGLVSPLLFFLVGSLYDRFGTRNIYYFGSLALαSPLFSVNWFLAVLGN
Rhizophydium_NC_003053.1_ND4  AYSSIAHγNVGMMGLFSNDINGITGAFIGSISHGFISGGLFIIIGMLYNRYGTKNINYYRGLVLFMPTFTFFFFIFTLSN
Hsapiens_NC_012920.1_ND4      AYSSISHMALVVTAILIQTPWSFTGAVILMIAHGLTSSLLFCLANSNYERTHSRIMILSQGLQTLLPLMAFWWLLASLAN
Dmelanogaster_NC_024511.2_ND4 AYSSVAHMGIVLSGLLTMTYWGLCGSYTLMIAHGLCSSGLFCLANVSYERLGSRSMLINKGLLNFMPSMTLWWFLLSSAN
Athaliana_NC_037304.1_ND4     AYSSVAHMNLVTIGMFSLNIQGIGGSILLMLSHGLVSSALFLCVGVLYDRHKTRLVRYYGGLVSTMPNFSTIFFFFTLAN
Gultimum_NC_014280.1_ND4      AYASVAHMNFIVLGIFSLTIQGLEGSIIQMISHGLISSGLFFSIGCLYDRYHTRFINYYGGLAHTMPLFCIALFIYVLAN
Jlibera_NC_021127.1_ND4       AYSSVAHMNYVTIGIFSQNIQGLQGSILLMLSHGIVSSALFMCIGVLYDRHKTRLIHYYSGLCQVMPVFATLFMVFIMAN
Lmarina_NC_045933.1_ND4       AYSSVAHMSVVMIGLFALTPQGLHGSLSLMLGHGIVSSALFLCVGVLYDRYHTRIIKYYGGLMQVMPNFSLFFLFFTLAN

Pboryanum_KR026342.1_ND4      MAMPLTPNFLAEFLCLCSIFAHNTIALAGALIAVILAAAYSMWAYARVVHGMPKPYVFEALTDLNRREFWTFVPLIFMTL
Pminor_NC_000892.1_ND4        VAFPGTGSFVGEαTIFISLLFMNPFCGαLLVFATFANLAWNFLLαTRVVFLHYQαNYLTFFYDISLKEFVIFLLLNILSI
Rhizophydium_NC_003053.1_ND4  LGFPFTMSFIGENLLFFSTINISPLITIFITSVSILLPIYFIYFYQRISYGKLSPYLLTLYQDLNIKEINLLLPLILLNT
Hsapiens_NC_012920.1_ND4      LALPPTINLLGELSVLVTTFSWSNITLLLTGLNMLVTALYSLYMFTTTQWGSL-THHINNMKPSFTRENTLMFMHLSPIL
Dmelanogaster_NC_024511.2_ND4 MAAPPTLNLLGEIYLLNSIVSWSWISMILLSFLSFFSAAYTLYLYSFSQHGKL-FSGVYSFSSGKIREYLLMLLHWLPLN
Athaliana_NC_037304.1_ND4     MSLPGTSSFIGEFLILVGAFQRNSLVATLAALGMILGAAYSLWLYNRVVSGNLKPDFLHKFSDLNGREVFIFIPFLVGVV
Gultimum_NC_014280.1_ND4      MSIPGSSSFVGELLILTGVFEDNTTTAIFATIGMFLGGVYSLLFYNRICYGNIQNVYLKVYYDLTYREFLIHLILIINIF
Jlibera_NC_021127.1_ND4       LSLPSTSSFVGEFLVLVGIFSSNTTVAFLATTGIILGAAYSLWLYNRVIFGSLKLNFASSFEDVTRREVYMLMPLVLLTF
Lmarina_NC_045933.1_ND4       IGFPGLCNFVGEFLCFLGAFERNALASFLCLMGTLGGVGYSMWLANRLVFGELRAKYITTFTDLSLVEYVMLVPFIFLTL

Pboryanum_KR026342.1_ND4      WLGLKPNVVLDSLSSSLIYWHQASLAVKDPASWALALL
Pminor_NC_000892.1_ND4        FSGLFAFELSSFLEYGFYYα------------------
Rhizophydium_NC_003053.1_ND4  IFGLFPYMVFETTMIPFLNVLNAN--------------
Hsapiens_NC_012920.1_ND4      LLSLNPDIITGFSS------------------------
Dmelanogaster_NC_024511.2_ND4 LLILKSESFMLWL-------------------------
Athaliana_NC_037304.1_ND4     WMGVYPKVFLDCMHTSV-----SNLVQHGKFH------
Gultimum_NC_014280.1_ND4      LLGLYPKIFESCMHESV-----SKILIHIDFSFFY---
Jlibera_NC_021127.1_ND4       LMGIYPSLFLDPMYTSL-----STYVGNLK--------
Lmarina_NC_045933.1_ND4       VIGVYPSIITDTADPAI-----FDLCVKLKVI------
```

Supplementary figure 3. Alignment of NADH dehydrogenase subunit 4 (ND4) predicted protein sequences from the three mitochondrial genomes with an unexpected Codetta inference and six reference species. In *Pseudopediastrum boryanum* (KR026342.1) and *Pedinomonas minor* (NC_000892.1), AUA codons are represented by alphas (α). In *Rhizophydium* sp. 136 (NC_003053.1), AAG codons are represented by gammas (γ). Yellow highlighted columns contain the codons under question and are conserved for the same amino acid in all six reference species. Triangles indicate positions associated with complex I diseases when mutated (from Bridges et al, 2011).

```
Pboryanum_KR026342.1_ND5      ---------MYLC-AVLAPFLGSALAGLFGRLLGSRGSGCVTILGLFTSMVFSFAIYYDISAMGAPVALDLGSW--FSAG
Pminor_NC_000892.1_ND5        ---------MYLI-ILLFPLISLFVαSαNLNFLGIRGFLYLSTFSLAVSLLLAHVαLYDVLILGαFTTVDLGEW--IYFY
Rhizophydium_NC_003053.1_ND5  ---------MYLT-IILLPLLSSIFST--NRKCGMIVGPILCILTMFFAAVFTLLLFYEVGINNSSVNLNLGSW--VDFG
Hsapiens_NC_012920.1_ND5      MTMHTTMTTLTLT-SLIPPILTTLVNPNKKNSYPHYVKSIVAS-----TFIISLFPTTMFMCLDQEVIISNWHW--ATTQ
Dmelanogaster_NC_024511.2_ND5 -----------MCSISFVNLISMS---------------LSCFLLSLYFLLNDMIYFI-------------EWELVSLN
Athaliana_NC_037304.1_ND5     ---------MYLL-IVFLPLLGSSVAGFFGRFLGSEGSAIMTTTCVSFSSILSLIAFYEVALGASACYLRIAPW--ISSE
Gultimum_NC_014280.1_ND5      ---------MLLLSIVFLPLLGSIASGLFGFYIGRKGSVFITTLTTFLSFIFSLIIFYDSITSEYEYLIYLATW--INSG
Jlibera_NC_021127.1_ND5       ---------MYLT-LLFLPILSSLTVLSFGRSIGKWGSCILSVASVVLCSIFSWICFYEVGLCGSVCRVELFPW--FHSE
Lmarina_NC_045933.1_ND5       ---------MYLL-VIFLPLLSAIICCFFSRWIGFLRAWQISTSLLGITFCLCLMIFYEIGLSSTLCTVEIGYW--FNIG
                                                                                 ▼
Pboryanum_KR026342.1_ND5      SVHVSWILNIDALSAVαMVTVSTVSFCVHVYSLGYMMADPHLPRFVSYLSLFTGSMLLLVTASDFVTMLVG-WEMIGVCS
Pminor_NC_000892.1_ND5        ELGSYFTFTFDVVSSIFIYVVLFISFLVHSYSIFYαSQDPYSSRFILYLNLFTWFαLVLVVSENALTLFLG-WEGIGITS
Rhizophydium_NC_003053.1_ND5  PILIDWSFIFDALTVSMYLPIVIITFLILIYSNEYLGHDPHKSRFFSLLSLFAFTMLLLVSGDNLLLMFAGLWEGVGIVS
Hsapiens_NC_012920.1_ND5      TTQLSLSFKLDYFSMMFIPVALFVTWSIMEFSLWYMNSDPNINQFFKYLLIFLITMLILVTANNLFQLFIG-WEGVGIMS
Dmelanogaster_NC_024511.2_ND5 SMSIVMTFLFDWMSLLFMSFVLMISSLVIFYSKEYMMNDNHINRFIMLVLMFVLSMMLLIISPNLISILLG-WDGLGLVS
Athaliana_NC_037304.1_ND5     MFDASWGFLFDSLTVVMLIVVTFISSLVHLYSISYMSEDPHSPRFMCYLSIFTFFMLMLVTGDNFLQLFLG-WEGVGLAS
Gultimum_NC_014280.1_ND5      LFNCNWSFLFDTLTMVMLVVVTSISTLVHLYSSQYMAHDPHLSRFMSYLSLFTFFMIILVTGDNFVQMFVG-WEGVGFCS
Jlibera_NC_021127.1_ND5       LVETNWGFLFDSLTVTMLIVVSTISSIVHIYSVGYMSHDPHLPRFMSYLSLFTFFMLILVTGDNFLQLFLG-WEGVGLCS
Lmarina_NC_045933.1_ND5       FFQSPWGFLFDSLTASMLIVVTSVSFLVHMYSGSYMEHDPHLGRFMGYLSLFTFFMLILVTGDNFMQLFLG-WEGVGLSS

Pboryanum_KR026342.1_ND5      YLLIGFWFHRLSATKAAQKAVLVNRVSDTGLLVGLMAAWWYLGSTDFSILYNTTTAC------AYA----------DLIC
Pminor_NC_000892.1_ND5        YLLINFWYSKLQSGKSAαKAVFLNRIGDSFFLIALGLTFYLFGSDDLFLISS---------LSVFY-----EKQIVKYLI
Rhizophydium_NC_003053.1_ND5  WALVNFWYTRIAANQASLSALFLNKIGDYGFILALVLSIALFSDLSLATIFN---------LGSYI-----NGDLLFLFT
Hsapiens_NC_012920.1_ND5      FLLISWWYARADANTAAIQAILYNRIGDIGFILALA----------WFILHSNSWDPQQM---ALL-----NANPSLTPL
Dmelanogaster_NC_024511.2_ND5 YCLV-IYFQNIKSYNAGMLTALSNRIGDVALLLSIA----------WMLNYG---------SWNYIFYLEIMQNEFEMLM
Athaliana_NC_037304.1_ND5     YLLIHFWFTRLQADKAAIKAMLVNRVGDFGLALGILGCFTLFQTVDFSTIFACASVPRN----SWI-FCNMRLNAISLIC
Gultimum_NC_014280.1_ND5      FLLINFWFTRLQANKAAIKAMLVNRISDLIFLLGILTIFYNIRTVEYFSTFAAISIVKDF---NFI-FFNYSLNIIDVAC
Jlibera_NC_021127.1_ND5       YLLINFWYTRLQANKSAIKAMIMNRLGDFGLSLGLLGIFYLFRSLDYDTVFSCAQEMSE----EYFGFLGIQMHALTVIC
Lmarina_NC_045933.1_ND5       YLLINFWFTRIQANKAAIKAMMVNRIGDISLTLGIALIYIYFKSVKYAVVFA---MAKEFEFSNFY-FLGFNWNTLDLIC
                                                      ▼▼     ▼      ▼  ▼
Pboryanum_KR026342.1_ND5      Y--TILIGALGKSAQVGLHVWLADAMEGPTPVSALIHAATLVTAGVYLIARTS--ALWECSTNART-LLLFVGAVTSLMA
Pminor_NC_000892.1_ND5        I--AYLIASαAKSAQILLHVWLPDAαEAPTPVSSLLHAATLVGAGVYLLIKLSFLSLLDFDSSN---FIαVIGILTSFLA
Rhizophydium_NC_003053.1_ND5  L--SILIAAMAKSAMIGLHSWLPKAγEGPTSVSALLHSSTMVTAGVFLLIRIS--PLLEFSSTTLM-IIIWLGSLGCLFG
Hsapiens_NC_012920.1_ND5      L--GLLLAAAGKSAQLGLHPWLPSAMEGPTPVSALLHSSTMVVAGIFLLIRFH--PLAENSPLIQT-LTLCLGAITTLFA
Dmelanogaster_NC_024511.2_ND5 IGSLVMLAAMTKSAQIPFSSWLPAAMAAPTPVSALVHSSTLVTAGVYLLIRFN--IILSTSWLGQ--LMLLLSGLTMFMA
Athaliana_NC_037304.1_ND5     I--LLFIGAVGKSAQIGLHTWLPDAMEGPTPVSALIHAATMVTAGVFMIARCS--PLFEYSPTALI-VITFAGAMTSFLA
Gultimum_NC_014280.1_ND5      I--LIFIGAMGKSAQIFLHLWLPDAMEGPTPVSALIHAATMVTAGVYLTARCS--PIFEYSIFSLK-VVTIIGASTAFFA
Jlibera_NC_021127.1_ND5       I--LLFVGAVGKSSQLGLHTWLPDAMEGPTPVSALIHAATMVTAGIFLVVRCS--PLFEYSSTALV-VITIVGSATAFFA
Lmarina_NC_045933.1_ND5       I--FLLIAAVGKSAQLGLHTWLPDAMEGPTPVSALIHAATMVTAGVFLLVRCS--ALYEHASENIRLLVVIVGALTAFFA

Pboryanum_KR026342.1_ND5      ATLGLVQNDLKRVIAYSTCSQLGYMMVSCGLSAYSLAIYHLLTHACFKALLFLGAGVAIHATADVQDLRRQGGAHQALPW
Pminor_NC_000892.1_ND5        GLIGFNQFDTKRIIAYSTCSQIGLMFYAIGLαSLDFSYLHFVIHGVFKCLMFLLAGAFIHTαFNEQDIRKYGSLNFLNSF
Rhizophydium_NC_003053.1_ND5  AATGLIINDLKGIIAYSTMSQLGYMIVAVGISQYNVALFHLITHAFFKSLLFLASGAVLHAVMDNQDIRKMGGLNIHLPY
Hsapiens_NC_012920.1_ND5      AVCALTQNDIKKIVAFSTSSQLGLMMVTIGINQPHLAFLHICTHAFFKAMLFMCSGSIIHNLNNEQDIRKMGGLLKTMPL
Dmelanogaster_NC_024511.2_ND5 GLGANFEFDLKKIIALSTLSQLGLMMSILSMGFLKLAMFHLLTHALFKALLFMCAGAIIHNMNNSQDIRLMGGLSIHMPL
Athaliana_NC_037304.1_ND5     ATTGILQNDLKRVIAYSTCSQLGYMIFACGISNYSVSVFHLMNHAFFKALLFLSAGSVIHAMSDEQDMRKMGGLASSFPL
Gultimum_NC_014280.1_ND5      STVGLVQNDFKKIVAYSTCSQLGYMFFACGLSNYPLAIFHLSNHAYFKALLFLCSGAVIHAMGDEQDIRKMGGLRRILPF
Jlibera_NC_021127.1_ND5       ATTGMVQNDIKRVIAYSTCSQLGYMAFACGLSGYSIGMFHLMNHAFFKALLFLGAGCVIHALADEQDMRKMGGLVRYLPF
Lmarina_NC_045933.1_ND5       ATTGLMQHDMKKVIAYSTCSQLGYMVFACGLSFYSVGMFHLINHAFFKALLFLSAGSVIHSLSDEQDIRQMGGLARLIPF
                                                      ▼
Pboryanum_KR026342.1_ND5      AWCCLFLGSLSLMGWPFLAGYYSKDAILELCWATTTPGGVYSYFILMTVAAFTSAYSFRVLIATFMAPSNSRHTEVSHT-
Pminor_NC_000892.1_ND5        LSLAFVLSNLSLLGαFFαAGFYSKELLL----αSGIYYNNFWSLLSVLAIFTTCLYGIKSILLVLSGSPNWNSFFSαTIY
Rhizophydium_NC_003053.1_ND5  TYLVFLFGSLSLMAFPFTAGFYSKDFLLEILLVPNNFTHTIAYIFTLLGALLTSTYSARTγMIALLSRPLFQKSIVHYVK
Hsapiens_NC_012920.1_ND5      TSTSLTIGSLALAGMPFLTGFYSKDHIIET--ANMSYTNAWALSITLIATSLTSAYSTRMILLTLTGQPRFPT--LTNIN
Dmelanogaster_NC_024511.2_ND5 TSACFNVSNLALCGMPFLAGFYSKDMILEI--VSISNVNMFSFFLYYFSTGLTVSYSFRLVYYSMTGDLNCGS--LNMLN
Athaliana_NC_037304.1_ND5     TYAMMLIGSLSLIGFPFLTGFYSKDVILELAYTKYTISGNFAFWLGSISVLFTSYYSFRLLFLTFLVPTNSFGRDISRCH
Gultimum_NC_014280.1_ND5      TYIMFLIGSLSLMGFPFLTGFYSKDLILEVSFASFSETGHFAYWLGTIGAFFTAFYSTRLLFFAFLSETNAYKNIIKHAH
Jlibera_NC_021127.1_ND5       TYGMMLLGSLSLMGFPFLTGFYSKDVILELAYAKFSMEGTFAHWLGTSSAFLTAFYSFRLLYYTFLSNPNGSKSIYLHAH
Lmarina_NC_045933.1_ND5       TYVFIMIGSLSLMGFPFLTGFYSKDLILELAFSTFTPVGHFAFWLGSLAAFFTALYSFRIIYYVFIGPINGNRVVYSNIH

Pboryanum_KR026342.1_ND5      GLPYTMGSSLVILAIGSIFAGYLFNDSLVGWGSGFFRTSIINGPATVQTVSSHM-MPVLYTYLPLLTVPIGFVLAY----
Pminor_NC_000892.1_ND5        DTNLαVIIILSVLVILNTFFGPMITEQ-IKLMDIVYNKSVFFSIKSNLFLFEHVYVNWIILFVISVFLILSWNYSIF---
Rhizophydium_NC_003053.1_ND5  DSGMFMTIPLLILSVAAVIIGYITNEIFITSAMPSYFNSIFIHPDNIRILDAPN-ANSIMSLIPLLFLFI-LFTALIIKS
Hsapiens_NC_012920.1_ND5      ENNPTLLNPIKRLAAGSLFAGFLITNN-ISPASPFQTT-----------------IPLYLKLTALAVTFLGLLTALDLN-
Dmelanogaster_NC_024511.2_ND5 DESWIMLRGMMGLLIMSIIGGSMLNWL------------IFPFPYMIC-------LPIYMKLLTLFVCIVGGLFGYLIS-
Athaliana_NC_037304.1_ND5     DAPIPMAIPSILLALGSLFVGYLAKDMMIGLGWNFWANSLLVLPKNEILAESEFAAPTIIKLIPILFSTLGAFVAYNVNL
Gultimum_NC_014280.1_ND5      DVPLEMGLPLGLLAFGSIFIGYLSKDMFVGLGSNFWNNSIYIDPINNQMIDAEF-LPTIFKLLPVILSFCGLFSAFYLYF
Jlibera_NC_021127.1_ND5       DAPAIMSIPLLVLAFGSIFVGYLAKDLMIGLGTDFWAGSIFVLPEHIIQLQAEF-IPSWIKMTPVFFSLLGASSAMLLYS
Lmarina_NC_045933.1_ND5       DAPTLIAIPLTILGFASIFAGYVGKDLFVGLGTDFWGNSIYCSSINTSTVDAEF-LPVFFKLIPVILSMCGVATA---YL

Pboryanum_KR026342.1_ND5      ---SFLWPLPYCADTGLRI---------------------------------VYKFLMARWHFDFVWNTHVAKPVLDLG-
Pminor_NC_000892.1_ND5        ---------FFSEKSLSFI------------------------NLKKI----PMLFFYNRGGFDAIY-QIISYKIFDFSY
Rhizophydium_NC_003053.1_ND5  PSLNNSSSIKDTANQISLNSTSNSSISNNHAISLINQEMSSPENNKVLIGVPMVNNLTILIHFN-IFNHWFMHRILILS-
Hsapiens_NC_012920.1_ND5      ---------YLT-NKLKMK---------------------SP--------LCTFYFSNMLGFYPSITHRTIPYLGLLTSQ
Dmelanogaster_NC_024511.2_ND5 -----LSNLFFLNKSLFMY------------------------N--------LSTFLGSMWFMPYISTYGMIFYPLNYG-
Athaliana_NC_037304.1_ND5     VADQFQRA-FQT-STFCNR---------------------------------LYSFFNKRWFFDQVLNDFLVRSFLRFGY
Gultimum_NC_014280.1_ND5      FKFRFLYN-LKT-SNYGLY---------------------------------FYNFLNRKWYFDKIYYEFINQYILKIGY
Jlibera_NC_021127.1_ND5       IGSSFSSTFFLT-NQVVRS---------------------------------IYLFLAKRWFFDIVYNELIAKKLLRVGY
Lmarina_NC_045933.1_ND5       FQTHSLFT-FKTESQVSVI---------------------------------LFNFLNRKWLFDRVYNEWVSQKGLWFGY

Pboryanum_KR026342.1_ND5      TYTWAIVDKGILEIL---GPRGLRNTILERAVPVVRAMQTGTVHDYALMLQIAVIAGLFAYALPN--------LYESMAS
Pminor_NC_000892.1_ND5        RVNWLANDKGYLPLFIFYFPKSVFSVVTNFNLα----MQHGLISIYYISIFSGLLFFILFI-------------------
Rhizophydium_NC_003053.1_ND5  NYLYRYIDKGILETI---GPTGFSRTFHFIGFLIEL-LATGNILNYALIIIFSILFII----------------------
Hsapiens_NC_012920.1_ND5      NLPLLLLDLTWLEKL---LPKTISQ--HQISTSIITSTQKGMIKLYFLSFFFPLILTLLLIT------------------
Dmelanogaster_NC_024511.2_ND5 QLVVKSFDQGWSEYF---GGQHLYQKLSMYSKTLFL-MHNNSLKIYLLLFVFWILILLILLFL-----------------
Athaliana_NC_037304.1_ND5     EVSFEALDKGAIEIL---GPYGISYTFRRLAERISQ-LQSGFVYHYAFAMLLGLTLFVTFFCMWDSLSSWV---DNRLSF
Gultimum_NC_014280.1_ND5      NFTYKMVDKGLIEMC---GPYGLITIFSFLSRQVVL-LQTGYIYHYSLLILISTIFLINIIF-------FSLLFYFNFII
Jlibera_NC_021127.1_ND5       HTTLVSLDKGVIELI---GPHGLEKAVVQISQGISK-LQSGYIYHYAFVMLIGITLLVSISAFWN----WIMIFDWRISL
Lmarina_NC_045933.1_ND5       VISYKTIDKGVLEFF---GPAGLTHLAKNLSRFSTN-LQTGLIFSYAFLVLLVVLLLLSL----------TLFYYYEIAI

Pboryanum_KR026342.1_ND5      SMAFARALTVALMLLVLAP---------------
Pminor_NC_000892.1_ND5        ----------------------------------
Rhizophydium_NC_003053.1_ND5  ILLF-----GFFI---------------------
Hsapiens_NC_012920.1_ND5      ----------------------------------
Dmelanogaster_NC_024511.2_ND5 ----------------------------------
Athaliana_NC_037304.1_ND5     ILIV----SSF--------YTKSSQE--------
Gultimum_NC_014280.1_ND5      IILF------FLIFLIIK----------------
Jlibera_NC_021127.1_ND5       IITL----TTLCLFFTWD-YKKEGQIESKTSLTN
Lmarina_NC_045933.1_ND5       IETFCIAISAFYIFLFVDRYVL------------
```

Supplementary figure 4. Alignment of NADH dehydrogenase subunit 5 (ND5) predicted protein sequences from the three mitochondrial genomes with an unexpected Codetta inference and six reference species. In *Pseudopediastrum boryanum* (KR026342.1) and *Pedinomonas minor* (NC_000892.1), AUA codons are represented by alphas (α). In *Rhizophydium* sp. 136 (NC_003053.1), AAG codons are represented by gammas (γ). Yellow highlighted columns contain the codons under question and are conserved for the same amino acid in all six reference species. Triangles indicate positions associated with complex I diseases when mutated (from Bridges et al, 2011).

References

1. Shulgina, Y. and Eddy, S.R. (2021), A computational screen for alternative genetic codes in over 250,000 genomes, *eLife* **10**, e71402.

2. Shen, X.X. et al (2018), Tempo and mode of genome evolution in the budding yeast subphylum, *Cell* **175**, 1533-1545.e20.

3. Mühlhausen, S. et al (2018), Endogenous stochastic decoding of the CUG codon by competing Ser- and Leu-tRNAs in *Ascoidea asiatica*, *Current Biology* **28**, 2046-2057.e5.

4. Dutilh, B.E. et al (2011), FACIL: Fast and accurate genetic code inference and logo, *Bioinformatics* **27**, 1929-1933.

5. Eddy, S.R. (2011), Accelerated profile HMM searches, *PLoS Computational Biology* **7**, e1002195.

6. Mistry, J. et al (2020), Pfam: The protein families database in 2021, *Nucleic Acids Research* **49**, D412-D419.

7. UniProt Consortium (2021), UniProt: The universal protein knowledgebase in 2021, *Nucleic Acids Research* **49**, D480-D489.

8. Kalvari, I. et al (2021), Rfam 14: Expanded coverage of metagenomic, viral and microRNA families, *Nucleic Acids Research* **49**, D192-D200.

9. Nawrocki, E.P. and Eddy, S.R. (2013), Infernal 1.1: 100-fold faster RNA homology searches, *Bioinformatics* **29**, 2933-2935.

10. Katoh, K. and Standley, D.M. (2013), MAFFT multiple sequence alignment software version 7: Improvements in performance and usability, *Molecular Biology and Evolution* **30**, 772-780.

11. Bridges, H.R. et al (2011), The mitochondrial-encoded subunits of respiratory complex I (NADH:ubiquinone oxidoreductase): Identifying residues important in mechanism and disease, *Biochemical Society Transactions* **39**, 799-806.

12. Lloyd, R.E. and McGeehan, J.E. (2013), Structural analysis of mitochondrial mutations reveals a role for bigenomic protein interactions in human disease, *PLoS One* **8**, e69003.
